# Kappa Free Light Chains, Soluble Interleukin-2 Receptor, and Interleukin-6 Help Explore Patients Presenting With Brain White Matter Hyperintensities

**DOI:** 10.3389/fimmu.2022.864133

**Published:** 2022-03-25

**Authors:** Michael Levraut, Cassandre Landes, Lydiane Mondot, Mikael Cohen, Saskia Bresch, Vesna Brglez, Barbara Seitz-Polski, Christine Lebrun-Frenay

**Affiliations:** ^1^URRIS-UR2CA, Centre Hospitalier Universitaire de Nice, Nice, France; ^2^Département de Médecine Interne, Centre Hospitalier Universitaire de Nice, Nice, France; ^3^Département de Neurologie, CRC SEP, Centre Hospitalier Universitaire de Nice, Nice, France; ^4^Département de Radiologie, Centre Hospitalier Universitaire de Nice, Nice, France; ^5^ImmunoPredict-UR2CA, Centre Hospitalier Universitaire de Nice, Nice, France; ^6^Laboratoire d’Immunologie, Centre Hospitalier Universitaire de Nice, Nice, France

**Keywords:** white matter hyperintensities, multiple sclerosis, biomarker, IL-6, sIL-2R, kappa free light chains

## Abstract

**Introduction:**

Many patients are referred to multiple sclerosis (MS) tertiary centers to manage brain white matter hyperintensities (WMH). Multiple diagnoses can match in such situations, and we lack proper tools to diagnose complex cases.

**Objective:**

This study aimed to prospectively analyze and correlate with the final diagnosis, cerebrospinal fluid (CSF) interleukin (IL)-1β, soluble IL-2 receptor (CD25), IL-6, IL-10, and kappa free light chains (KFLC) concentrations in patients presenting with brain WMH.

**Methods:**

All patients over 18 years addressed to our MS tertiary center for the diagnostic workup of brain WMH were included from June 1, 2020, to June 1, 2021. Patients were separated into three groups—MS and related disorder (MSARD), other inflammatory neurological disorder (OIND), and non-inflammatory neurological disorder (NIND) groups—according to clinical presentation, MRI characteristics, and biological workup.

**Results:**

A total of 176 patients (129 women, mean age 45.8 ± 14.7 years) were included. The diagnosis was MSARD (*n* = 88), OIND (*n* = 35), and NIND (*n* = 53). Median CSF KFLC index and KFLC intrathecal fraction (IF) were higher in MSARD than in the OIND and NIND groups; *p* < 0.001 for all comparisons. CSF CD25 and IL-6 concentrations were higher in the OIND group than in both the MSARD and NIND groups; *p* < 0.001 for all comparisons. KFLC index could rule in MSARD when compared to NIND (sensitivity, 0.76; specificity, 0.91) or OIND (sensitivity, 0.73; specificity, 0.76). These results were similar to those with oligoclonal bands (sensitivity, 0.59; specificity, 0.98 compared to NIND; sensitivity, 0.59; specificity, 0.88 compared to OIND). In contrast, elevated CSF CD25 and IL-6 could rule out MSARD when compared to OIND (sensitivity, 0.58 and 0.88; specificity, 0.95 and 0.74, respectively).

**Discussion:**

Our results show that, as OCBs, KFLC biomarkers are helpful tools to rule in MSARD, whereas elevated CSF CD25 and IL-6 rule out MSARD. Interestingly, CSF IL-6 concentration could help identify neuromyelitis optica spectrum disorder, myelin oligodendrocyte glycoprotein antibody-associated disease, and central nervous system (CNS) vasculitis. These results need to be confirmed within more extensive and multicentric studies. Still, they sustain that KFLC, CSF CD25, and CSF IL-6 could be reliable biomarkers in brain WMH diagnostic workup for differentiating MSARD from other brain inflammatory MS mimickers.

## Introduction

Multiple sclerosis (MS) is a chronic inflammatory and demyelinating central nervous system (CNS) disease. It presents as relapsing clinical demyelinating events or a progressive worsening neurological deficit disease with suggestive white matter hyperintensities on the brain or spinal cord MRI T2-weighted images. Clinical research has focused on diagnosing MS as early as possible to prevent relapse and disability by initiating disease-modifying treatments. In this condition, many patients may have an early demyelinating disease diagnosis: i) after a single demyelinating event (1, 2) or ii) before any clinical event (3, 4). In early-MS patients, biology may have an essential role in identifying an intrathecal B-cell activation by the detection of cerebrospinal fluid (CSF) oligoclonal bands (OCBs) on isoelectric focusing, which can replace dissemination in time in patients presenting with a typical first demyelinating event (1, 2). Unfortunately, misdiagnosis may occur in such situations (5, 6), while many other neurological diseases may mimic early MS (6, 7).

The immunopathology of MS is complex and implicates a large number of cells. CD4+ Th1 and Th17 cells are thought to promote while CD4+ Th2 and Treg cells are thought to downregulate inflammation in MS (8–10). B cells are also crucial effector cells in MS (11). In contrast, i) B cell-depletive therapies are effective in relapsing MS (12, 13), and ii) intrathecal immunoglobulin synthesis is part of the MS diagnostic criteria (1). However, we lack a reliable biomarker that could help separate MS from other inflammatory-mimicking diseases to avoid misdiagnosis.

During the last decade, many biomarkers have been explored. Kappa free light chains (KFLC), low-weighted immunoglobulin compounds, are a reliable biomarker in MS (14–16). This activated B-cell biomarker has the advantage, compared to OCBs, to quantify intrathecal B-cell activity by an automatized procedure. However, prospective data on the effectiveness of KFLC biomarkers are poor (14). Cytokines are low-molecular-weight proteins secreted by many cells, implicated in many immune functions, such as chemotaxis, activation, or repression of the immune cells. In autoimmune CNS diseases, cytokine measurement may reflect a unique immunopathological profile and help etiological diagnosis. It has been shown that CSF interleukin (IL)-6 is increased in neuromyelitis optica spectrum disorders (NMOSD) or in myelin oligodendrocyte glycoprotein antibody-associated disease (MOGAD) compared to MS (17). Soluble IL-2 receptor (s-IL2R), also called CD25, is increased in many CNS granulomatosis, such as neurosarcoidosis or in infectious meningitis (18), and CSF IL-10 is now part of the diagnostic workup in CNS lymphoma (19). However, our knowledge about cytokine expression in such diseases comes from retrospective cohorts. Based on these data, our MS tertiary center included OCBs, KFLC, and CSF IL-1β, sIL-2R, IL-6, and IL-10 concentration measurement in the routine diagnostic workup of patients presenting with white matter hyperintensities suggestive of MS.

Therefore, in this study, we prospectively evaluated the expression of KFLC biomarkers and CSF concentration of IL-1β, sIL-2R (CD25), IL-6, and IL-10, and we correlated each biomarker measurement with diagnosis in patients referred to our MS center for suspected MS.

## Methods

### Patients

All patients referred to our MS tertiary center in the University Hospital of Nice, France, were eligible for the study from June 1, 2020, to June 1, 2021. Patients were included if they i) were at least 18 years old and ii) had brain white matter hyperintensities on MRI T2-weighted images. According to routine care, all patients underwent the same diagnostic workup with a blood and CSF analysis and 3-T brain MRI.

At the end of the diagnostic workup, patients were separated into three groups according to their diagnosis. First, patients were divided as having an inflammatory or a non-inflammatory CNS disorder according to clinical presentation, MRI (topography, number, size, and gadolinium enhancement of the lesions), and biology (identification of blood or CSF red flags for MS). All non-inflammatory diagnoses were pooled together as a control group named non-inflammatory neurological disorder (NIND). Patients identified as having an inflammatory CNS disorder were separated into two groups. Patients who fulfilled the 2017 McDonald criteria for MS and clinically isolated syndrome (CIS) (1), or the 2009 criteria for radiologically isolated syndrome (RIS) (3, 20), were pooled together into the MS and related disorder (MSARD) group. The other inflammatory patients were pooled together as having another inflammatory disease: the other inflammatory neurological disease (OIND) group.

A non-opposition to research was obtained for each patient according to French law. Our institutional review board approved the study design, and the study was registered on Clinical Trial (NCT05056740) as the CyBIRD (Cytokine and Brain Inflammatory Related Disorders) Study.

### Kappa Free Light Chains and Cytokine Measurement

Blood and CSF were collected the same day for all patients. Fluids were sent within 2 h after collection into the Immunology Laboratory of Nice’s University Hospital.

Detection of OCBs was performed by isoelectric focusing and subsequent immunoglobulin using IgG-specific antibody staining. OCB patterns were evaluated by experienced biologists and classified as negative or positive. A cutoff ≥2 CSF-restricted bands was used to define OCB positivity. CSF KFLC was measured on fresh samples using the turbidimetric analyzer Optilite^®^ (The Binding Site, Birmingham, UK) with the serum-free light chain immunoassay Freelite^®^ (The Binding Site, Birmingham, UK). Serum and CSF albumin were also measured with the same turbidimetric analyzer and permitted to calculate KFLC index and KFLC intrathecal fraction (KFLC IF) as follows:

(i) KFLC index = KFLC quotient/albumin quotient with:


$$\begin{array}{l} KFLCquotient=CSFKFLC/serumKFLC\\ Albuminquotient=CSFalbumin/serumalbumin \end{array}$$

(ii) KLC IF was determined with Reiber’s formula (21):


$$\begin{array}{l} KFLCIF\left(\%\right)=\;\left(KFLC_{loc}/CSFKFLC\right)\;\times \;100\;with\\ KFLC_{loc}=\;\left(KFLCquotient/KFLCquotient\left(\lim\right)\right)\;\times \;serum\;KFLC\;with\\ KFLCquotient\left(\lim\right)\;=\;3.27\;{\left(albuminquotient^{2}+33\right)}^{0.5}\;-\;8.2\;\left(\times 10^{-3}\right) \end{array}$$

The turbidimetric analyzer’s lower detection limit (LDL) for KFLC was 0.33 mg/L. For patients with CSF KFLC concentration lower than the LDL, an empirical value of KFLC = LDL/2 = 0.16 mg/L was assigned.

For cytokine measurement, CSF was directly centrifuged and kept frozen at −80°C until there were enough stored samples to perform analysis (16-well cartridges). CSF was thawed once, just before cytokine analysis. CSF IL-1β, sIL-2R (or CD25), IL-6, and IL-10 concentration were determined using a mixture of 25 μl of CSF and buffer, in a custom-designed cartridge Ella (ProteinSimple, Santa Clara, CA, USA) for the detection of IL-1β, sIL-2R, IL-6, and IL-10, according to the manufacturer’s instructions. The LDL of ELISA kits for cytokine measurement was 0.32 pg/ml for IL-1β, 6.56 pg/ml for sIL-2R, 0.5 pg/ml for IL-6, and 1.16 pg/ml for IL-10. As for CSF KFLC measurement, when the CSF cytokine concentration was under the LDL, an empirical CSF cytokine value of LDL/2 was assigned.

### Statistical Analysis

Statistical analysis was performed using the online application EasyMedStat (version 3.14; www.easymedstat.com).

Data were presented as means with their SD for continuous values and counts and percentages for categorical variables for descriptive statistics. The data’s normality and heteroscedasticity were assessed using the Shapiro–Wilk and Levene’s tests. Constant values were compared using the Mann–Whitney U test. When more than two groups needed to be compared, the Kruskal–Wallis test was performed with a *post hoc* Conover’s multiple comparison test. Categorical variables were compared using the chi-square test. Receiver operating characteristic (ROC) curves were used to assess the ability of each biomarker to predict MSARD diagnosis and to calculate the area under the curve (AUC). DeLong’s test was performed to make pairwise comparisons of the predictive biomarkers according to MSARD diagnosis. The test implementation follows “Fast Implementation of DeLong’s Algorithm for Comparing the Areas Under Correlated Receiver Operating Characteristic Curves, by Xu Sun and Weichao Xu.” An optimal threshold that best discriminates MSARD from control populations was then determined with Youden’s index. Based on the defined threshold values, patients were classified as positive or negative for each biomarker as a binary result. Sensitivity, specificity, and positive and negative predictive values were then calculated for each biomarker. All comparisons were two-tailed. To identify the impact of demographic and clinical features on each biomarker concentration, CSF KFLC, IL-6, and CD25 were included in a multivariate linear regression model. According to the three identified groups, the explanatory variables were age, gender, disease duration, immune-modifying drug use at sampling, and final diagnosis. Data were checked for multicollinearity with the Belsley–Kuh–Welsch test. Patients with missing data were excluded from the analysis. The differences were considered significant when the *p*-value was <0.05.

## Results

### Study Cohort

In the study period, two hundred seventeen patients have been referred to our center for brain white matter T2 hyperintensities. Forty-one patients were excluded because of subnormal brain MRI or age <18 years. One hundred seventy-six patients were included in the study. After the diagnostic workup, patients were separated into the following groups: 88 patients (50%) in the MSARD group, 35 (20%) in the OIND group, and 53 (30%) in the NIND group (flowchart available in the **Supplementary Material**, **Figure S1**). MSARD patients were younger than OIND (*p* = 0.002) and NIND patients (*p* = 0.001). All MSARD patients, except for RIS, experienced a clinical demyelinating event, while 63% and 0% in the OIND and NIND groups, respectively, experienced the same (*p* < 0.001 for both comparisons). All groups were comparable for immune treatment exposure at sampling. MSARD patients had lower CSF protein level (*p* < 0.001), CSF white blood cell count (*p* < 0.001), and albumin quotient (*p* < 0.001) than had OIND patients but had a higher level of CSF immunoglobulin G (IgG) and positive OCB status (*p* = 0.005 and *p* < 0.001, respectively). All characteristics are shown in **Table 1**.

**Table 1:** Baseline demographic and clinical data.

	MSARD group *n* = 88	OIND group* n* = 35	*p*-Value MSARD vs. OIND	NIND group* n* = 53	*p*-Value MSARD vs. NIND
Median age, [IQR]	41.6 ± 13.0	50.7 ± 17.0	0.002	49.5 ± 13.5	0.001
Female gender, *n* (%)	67 (76)	19 (54)	0.028	43 (81)	0.535
Type of disease, *n* (%)	MS, 58 (66) CIS, 22 (25) RIS, 8 (9)	NMOSD/MOGAD, 9 (26) CNS vasculitis, 9 (26) CNS lymphoma, 3 (9) Immune encephalitis, 2 (6) CNS infection, 2 (6) Neurosarcoidosis, 2 (6) Other, 8 (23)	–	Migraine, 13 (25) SCVD, 18 (34) Stroke, 3 (6) Ischemic ON, 1 (2) Myelopathy, 3 (6) Cerebellar atrophy, 2 (4) Mechanical, 6 (11) Other, 7 (13)	–
Clinical event, *n* (%)	80 (91)	22 (63).	<0.001	0* (0)	<0.001
Optic neuritis, *n* (%)	7 (8)	4 (11)	–	0 (0)	
Myelitis, *n* (%)	41 (47)	8 (23)	–	0 (0)	
Brainstem/cerebellar, *n* (%)	20 (23)	4 (11)	–	0 (0)	
Other, *n* (%)	12 (13	6 (16)	–	0 (0)	
Autoimmune medical history, *n* (%)	14 (16)	6 (17)	1	16 (30)	0.056
Immune-modifying drug at sampling, *n* (%)	8 (9)	5 (15)	0.349	6 (11)	0.773
Gadolinium enhancement on baseline MRI, *n* (%)	28 (33)	21 (60)	0.008	2 (4)	<0.001
Median disease duration (months), [IQR]	5.3 [1.3; 35.5]	1.3 [0.3; 2.9]	<0.001	12.3 [3.8; 19.5]	0.108
Median CSF protein concentration (g/L), [IQR]	0.33 [0.27; 0.40]	0.45 [0.31; 0.94]	<0.001	0.33 [0.26; 0.48]	0.667
Median CSF WBC count (/µl), [IQR]	2 [0; 5]	2 [0; 25]	<0.001	0 [0; 1]	0.015
Median albumin quotient (%), [IQR]	0.44 [0.33; 0.58]	0.71 [0.51; 1.45]	<0.001	0.47 [0.36; 0.66]	0.308
Median IgG index, [IQR]	0.75 [0.61; 0.99]	0.60 [0.50; 0.71]	0.005	0.56 [0.50; 0.61]	<0.001
Median serum KFLC (mg/L), [IQR]	13.8 [11.7; 16.2]	15.0 [11.5; 19.6]	0.017	13.7 [11.2; 16.8]	0.258
Positive OCBs status, *n* (%)	52 (60)	4 (11)	<0.001	1 (2)	<0.001

CIS, clinically isolated syndrome; CNS, the central nervous system; CSF, cerebrospinal fluid; IQR, interquartile range; KFLC, kappa free light chains; MOGAD, myelin oligodendrocyte glycoprotein antibody-associated disease; MS, multiple sclerosis; MSARD, multiple sclerosis and related disorder; NIND, non-inflammatory neurological disorder; NMOSD, neuromyelitis optica spectrum disorder; OCBs, oligoclonal bands; OIND, other inflammatory neurological disorder; ON, optic neuritis; RIS, radiologically isolated syndrome; SCVD, small cerebral vessel disease; WBC, white blood cell.

*Clinical event non-evocative of demyelinating events (optic neuritis presented as an acute and non-painful event, myelopathies presented as progressive motor weakness of lower limbs).

### Quantification of Kappa Free Light Chains Biomarkers

**Figure 1:**
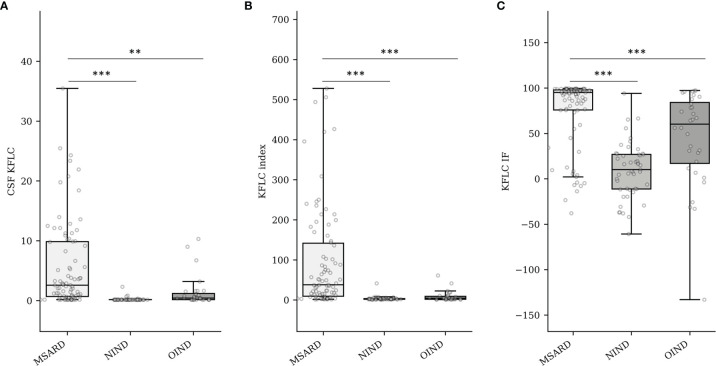
Quantification of CSF KFLC (mg/L) **(A)**, KFLC index **(B)**, and KFLC IF (%) into groups **(C)**. MSARD, multiple sclerosis and related disorder (*n* = 88); NIND, non inflammatory neurological disorder (*n* = 53); OIND, other inflammatory neurological disorder (*n* = 35). ** determined a *p*-value < 0.01. *** determined a *p*-value < 0.001. CSF, cerebrospinal fluid; KFLC, kappa free light chains; IF, intrathecal fraction.

Median values of CSF KFLC (**Figure 1A**), KFLC index (**Figure 1B**), and KFLC IF (**Figure 1C**) were higher in the MSARD group (2.59 (IQR 9.18) mg/L; 37.80 (IQR 132.07); 95.06% (IQR 22.09%), respectively) than in the NIND group (0.16 (IQR 0.06) mg/L, *p* < 0.001 for CSF KFLC; 2.38 (IQR 1.82), *p* < 0.001 for KFLC index; 10.11% (IQR 38.10%), *p* < 0.001 for KFLC IF) and the OIND group (0.43 (IQR 1.03) mg/L, *p* = 0.001 for CSF KFLC; 4.53 (IQR 7.35), *p* < 0.001 for KFLC index; 60.21% (IQR 66.98%), *p* < 0.001 for KFLC IF).

In the MSARD group, median values of CSF KFLC (figure in the **Supplementary Material**, **Figure S2A**), KFLC index (**Figure S2B**), and KFLC IF (**Figure S2C**) were lower in CIS (0.63 (IQR 1.64) mg/L, 14.2 (IQR 22.2), and 75.1% (IQR 87.19%), respectively) than in MS patients (3.8 (IQR 10.1) mg/L, 69.1 (IQR 161.23), and 96.6% (IQR 11.1%), respectively; *p* < 0.001 for all comparisons). There was no difference of KFLC biomarkers values between MS and RIS patients (3.4 (IQR 11.2) mg/L, 48.5 (IQR 213.6), and 94.2% (IQR 73.9%), for CSF KFLC (*p* = 0.403), KFLC index (*p* = 0.377), and KFLC IF (*p* = 0.320), respectively).

### Quantification of Cerebrospinal Fluid IL-1β, CD25, IL-6, and IL-10

**Figure 2:**
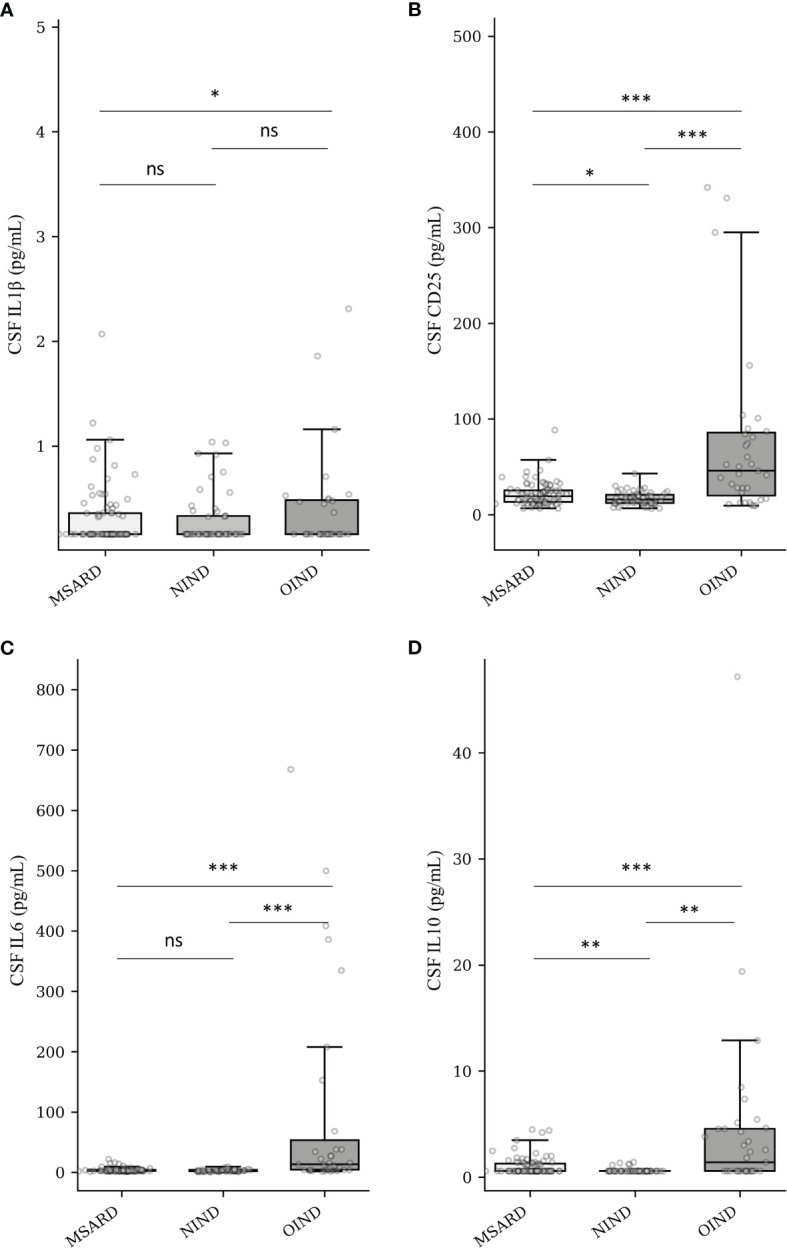
Quantification of CSF IL-1β **(A)**, CD25 (sIL-2R) **(B)**, IL-6 **(C)**, and IL-10 **(D)** into groups. MSARD, multiple sclerosis and related disorders (*n* = 88); NIND, non-inflammatory neurological disorders (*n* = 53); OIND, other inflammatory neurological disorders (*n* = 35); ns, non-significant (*p* > 0.05). *p < 0.05. **p < 0.01. ***p < 0.001. CSF, cerebrospinal fluid.

CSF concentration of IL-1β was often under the LDL of the analyzer (68% of the all cohort). Therefore, median values of CSF IL-1β (**Figure 2A**) were similar between groups: 0.16 (IQR 0.20) pg/ml in the MSARD group, 0.16 (IQR 0.17) pg/ml in the NIND group, and 0.16 (IQR 0.32) pg/ml in the OIND group. Median values of CSF CD25 (**Figure 2B**) were higher in the OIND group (45.9 (IQR 65.75) pg/ml) compared to the MSARD group (19.35 (IQR 12.12) pg/ml, *p* < 0.001), and the NIND group (15.7 (IQR 8.60) pg/ml, *p* < 0.001). Similar to CSF CD25, median values of CSF IL-6 (**Figure 2C**) were higher in the OIND group (13.6 (IQR 48.90) pg/ml) compared to the MSARD group (2.99 (IQR 1.67) pg/ml, *p* < 0.001), and the NIND group (2.68 (IQR 2.07) pg/ml, *p* < 0.001). CSF IL-10 concentration was under the LDL in most of the MSARD patients (67%) and the NIND patients (91%). Median values of CSF IL-10 (**Figure 2D**) were higher in the OIND group (1.40 (IQR 3.99) pg/ml) compared to the MSARD group (0.58 (IQR 0.69) pg/ml, *p* < 0.001) and the NIND group (0.58 (IQR 0.1) pg/ml, *p* = 0.002).

In the MSARD group, median values of CSF CD25 (**Figure S3A**) were higher in MS (20.5 (IQR 16.3) pg/ml) than in CIS patients (14.6 (IQR 11.4) pg/ml), *p* = 0.023. CSF CD25 median values were similar between MS and RIS patients (20.3 (IQR 7.9) pg/ml), *p* = 0.836. Median values of CSF IL-6 (**Figure S3B**) and IL-10 (**Figure S3C**) were similar between MS (3.1 (IQR 1.6) and 0.58 (IQR 0.8) pg/ml, respectively), RIS (2.5 (IQR 1.7) and 0.58 (IQR 0.2) pg/ml, respectively), and CIS patients (2.9 (IQR 2.1) and 0.58 (IQR 0.1) pg/ml, respectively), *p* > 0.1 for all comparisons.

### Biomarker Diagnostic Performances

We analyzed the ability of the KFLC index, KFLC IF, CSF CD25, CSF IL-6, and CSF IL-10 to diagnose MSARD i) against a non-inflammatory-mimicking disease by the comparison of the MSARD and NIND groups and ii) against another inflammatory-mimicking disease by the comparison of the MSARD and OIND groups. CSF IL-1β was not analyzed because of its low CSF concentration in most patients.

#### Kappa Free Light Chains Biomarkers Performed Better Than Cerebrospinal Fluid CD25, IL-6, and IL-10 in Separating Multiple Sclerosis and Related Disorder From Non-Inflammatory Neurological Disorder

KFLC index and KFLC IF had similar, and good, overall performances (AUC, 0.900 [0.849; 0.952] and 0.887 [0.830; 0.943], respectively) to diagnose MSARD compared to NIND. However, CSF CD25, CSF IL-6, and CSF IL-10 had lower performances (AUC, 0.596 [0.501; 0.690], 0.569 [0.467; 0.671], and 0.627 [0.565; 0.688], respectively) than both KFLC biomarkers (*p* < 0.001 for all comparisons). The thresholds that best separated MSARD from NIND were 8.4 for KFLC index, 73.1% for KFLC IF, 21.5 pg/ml for CSF CD25, 2.0 pg/ml for CSF IL-6, and 1.2 pg/ml for CSF IL-10. All data are shown in **Table 2**, and the ROC curves are available in the **Supplementary Material** (**Figure S4A**).

**Table 2:** Diagnostic performances of the different biomarkers for MSARD diagnosis compared to both control populations.

	MSARD vs. NIND* n* = 141			MSARD vs. OIND* n* = 123		
	AUC (%)	95% CI	Optimal threshold	AUC (%)	95% CI	Optimal threshold
KFLC index	90.0^a^	[84.9; 95.2]	8.4	82.3	[74.6; 90.0]	13.1
KFLC IF	88.7^a^	[83.0; 94.3]	73.1	74.5^c^	[65.2; 83.8]	82.8
CSF CD25	59.6^b^	[50.1; 69.0]	21.5	77.0^d^	[65.6; 88.5]	41.5
CSF IL-6	56.9^b^	[46.7; 67.1]	2.0	87.4^e^	[79.8; 95.0]	4.1
CSF IL-10	62.7^b^	[56.5; 68.8]	1.2	68.0	[56.6; 79.4]	2.4

CSF, cerebrospinal fluid; KFLC, kappa free light chains; MSARD, multiple sclerosis and related disorder; NIND, non-inflammatory neurological disorder; OIND, other inflammatory neurological disorder; AUC, area under the curve.

a KFLC index and KFLC IF AUC are not statistically different (p = 0.404).

b AUCs of CSF CD25, IL-6, and IL-10 were all lower than both KFLC biomarkers (p < 0.001 for all 6 comparisons). There was no AUC difference between CSF CD25 and CSF IL-6 (p = 0.717), CSF CD25 and CSF IL-10 (p = 0.523), and CSF IL-6 and CSF IL-10 (p = 0.314).

c KFLC index and KFLC IF AUC are statistically different (p = 0.008).

d CSF CD25 AUC is not statistically different than KFLC index AUC (p = 0.358), CSF IL-6 AUC (p = 0.119), or CSF IL-10 AUC (p = 0.149).

e CSF IL-6 AUC is not statistically different than KFLC index AUC (p = 0.436) and CSF CD25 AUC (p = 0.119) and is higher than CSF IL-10 AUC (p = 0.007).

These cutoffs, KFLC index, KFLC IF, CSF CD25, IL-6, and IL-10 were changed into binary variables, and patients were categorized as positive or negative for each biomarker. As shown in **Table 3**, both OCBs and the KFLC index had good overall performances for MSARD diagnosis as compared to NIND. OCBs were more specific than the KFLC index (0.98 vs. 0.91, respectively) but less sensitive (0.59 vs. 0.76, respectively). However, the KFLC index diagnostic accuracy seemed to be higher than OCBs’ (0.82 vs. 0.74). Interestingly, the combination of an elevated KFLC index and CSF IL-6 had the same specificity for MSARD diagnosis than OCBs (specificity of 0.96 vs. 0.98, respectively) with a higher sensitivity (0.69 vs. 0.59, respectively) and higher diagnostic accuracy (0.79 vs. 0.74).

**Table 3:** Diagnostic performance of the different biomarkers comparing MSARD to NIND (*n* = 141).

	TP (*n*)	FP (*n*)	TN (*n*)	FN (*n*)	Sensitivity (%)	Specificity (%)	PPV (%)	NPV (%)	Accuracy (%)
Positive OCBs	52	1	52	36	59.1 [48.1; 69.5]	98.1 [89.9; 99.9]	98.1 [88.1; 99.7]	59.1 [52.8; 65.1]	73.8 [65.7; 80.8]
KFLCi > 8.4	67	5	48	21	76.1 [65.9; 84.6]	90.6 [79.3; 96.9]	93.1 [85.2; 96.9]	69.6 [60.9; 77.0]	81.6 [74.2; 87.6]
CD25 > 21.5	37	13	40	51	42.0 [31.6; 53.0]	75.5 [61.7; 86.2]	74.0 [62.6; 82.9]	44.0 [38.3; 49.8]	54.6 [46.0; 63.0]
IL-6 > 2.0	79	37	16	9	89.8 [81.5; 95.2]	30.2 [18.3; 44.3]	68.1 [63.8; 72.1]	64.0 [45.8; 78.9]	67.4 [59.0; 75.0]
CD25 > 21.5 and IL-6 > 2.0	31	8	45	57	35.2 [25.3; 46.1]	84.9 [72.4; 93.3]	79.5 [65.8; 88.6]	44.1 [39.5; 48.9]	53.9 [45.3; 62.3]
KFLCi > 8.4 and CD25 > 21.5	29	0	53	59	33.0 [23.3; 43.8]	100.0 [93.3; 100.0]	100.0 [–]	47.3 [43.7; 51.0]	58.2 [49.6; 66.4]
KFLCi > 8.4 and IL-6 > 2.0	60	2	51	28	68.2 [57.4; 77.7]	96.2 [87.0; 99.5]	96.8 [88.4; 99.2]	64.6 [57.2; 71.3]	78.7 [71.0; 85.2]
KFLCi > 8.4 and CD25 > 21.5 and IL-6 > 2.0	25	0	53	63	28.4 [19.3; 39.0]	100.0 [93.3; 100.0]	100.0 [–]	45.7 [42.4; 49.0]	55.3 [46.7; 63.7]

FN, false negative; FP, false positive; KFLCi, kappa free light chains index; NPV, negative predictive value; PPV, positive predictive value; TN, true negative; TP, true positive; MSARD, multiple sclerosis and related disorder; NIND, non-inflammatory neurological disorder.

#### Cerebrospinal Fluid IL-6, CD25, and Kappa Free Light Chains Index Showed Good Performances in Diagnosing Multiple Sclerosis and Related Disorder Compared to Other Inflammatory Neurological Disorder

When comparing MSARD to OIND, KFLC index showed better diagnostic performances than KFLC IF (AUC, 0.823 [0.746; 0.900], and 0.745 [0.652; 0.838] respectively, *p* = 0.008). In contrast with the comparison with the NIND group, in this situation, CSF CD25 and CSF IL-6 showed good diagnostic performances (AUC, 0.770 [0.656; 0.885], and 0.874 [0.798; 0.950], respectively), statistically similar to the KFLC index (*p* = 0.358, and *p* = 0.436, respectively). However, diagnostic performances of CSF IL-10 (AUC, 0.680 [0.566; 0.794]) were lower than those of both KFLC index (*p* = 0.02) and IL-6 (*p* < 0.001). The thresholds that best separated MSARD from OIND were 13.1 for KFLC index, 82.8% for KFLC IF, 41.5 pg/ml for CSF CD25, 4.1 pg/ml for CSF IL-6, and 2.4 pg/ml for CSF IL-10. All data are shown in **Table 2**, and the ROC curves are available in the **Supplementary Material** (**Figure S4B**).

As shown in **Table 4**, CSF IL-6 could separate both groups with better sensitivity and the same specificity than OCBs and a better specificity for the same sensitivity than the KFLC index (sensitivity of 0.74, 0.59, and 0.73 and specificity of 0.88, 0.88, and 0.76 for CSF IL-6, OCBs, and KFLC index, respectively). The better specific combination for MSARD diagnosis in such a situation was the association of low CSF IL-6 and CD25 (sensitivity 0.72, specificity 0.94).

**Table 4:** Diagnostic performance of the different biomarkers comparing MSARD to OIND (*n* = 123).

	TP (*n*)	FP (*n*)	TN (*n*)	FN (*n*)	Sensitivity (%)	Specificity (%)	PPV (%)	NPV (%)	Accuracy (%)
Positive OCBs	52	4	29	36	59.1 [48.1; 69.5]	87.9 [71.8; 96.6]	92.9 [83.6; 97.1]	44.6 [37.8; 51.6]	66.9 [57.8; 75.2]
KFLCi > 13.1	64	8	25	24	72.7 [62.2; 81.7]	75.8 [57.7; 88.9]	88.9 [81.2; 93.7]	51.0 [41.3; 60.7]	73.6 [64.8; 81.2]
CD25 < 41.5	84	14	19	4	95.5 [88.8; 98.7]	57.6 [39.2; 74.5]	85.7 [80.1; 90.0]	82.6 [63.6; 92.8]	85.1 [77.5; 90.9]
IL-6 < 4.1	65	4	29	23	73.9 [63.4; 82.7]	87.9 [71.8; 96.6]	94.2 [86.5; 97.6]	55.8 [46.5; 64.7]	77.7 [69.2; 84.8]
CD25 < 41.5 and IL-6 < 4.1	63	2	31	25	71.6 [61.0; 80.7]	93.9 [79.8; 99.3]	96.9 [89.1; 99.2]	55.4 [46.8; 63.6]	77.7 [69.2; 84.8]
KFLCi > 13.1 and CD25 < 41.5	61	3	30	27	69.3 [58.6; 78.7]	90.9 [75.7; 98.1]	95.3 [87.3; 98.4]	52.6 [44.4; 60.8]	75.2 [66.5; 82.6]
KFLCi > 13.1 and IL-6 < 4.1	48	2	31	40	54.5 [43.6; 65.2]	93.9 [79.8; 99.3]	96.0 [86.1; 98.9]	43.7 [37.8; 49.7]	65.3 [56.1; 73.7]
KFLCi > 13.1 and CD25 < 41.5 and IL-6 < 4.1	46	0	33	42	52.3 [41.4; 63.0]	100.0 [89.4; 100.0]	100.0 [-]	44.0 [38.7; 49.4]	65.3 [56.1; 73.7]

FN, false negative; FP, false positive; KFLCi, kappa free light chains index; NPV, negative predictive value; PPV, positive predictive value; TN, true negative; TP, true positive; MSARD, multiple sclerosis and related disorder; OIND, other inflammatory neurological disorder.

#### Kappa Free Light Chains Index, Cerebrospinal Fluid CD25, and Cerebrospinal Fluid IL-6 Diagnostic Performances Needed to Be Studied in Homogenized Other Inflammatory Neurological Disorder Populations

**Figure 3:**
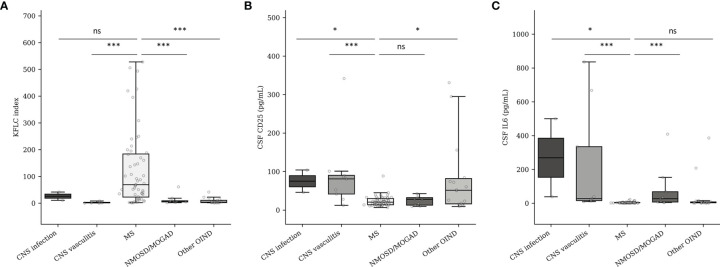
KFLC index **(A)**, CSF CD25 **(B)**, and CSF IL-6 **(C)** expression in MS and OIND subgroups. CNS, central nervous system; MS, multiple sclerosis; MOGAD, myelin oligodendrocyte antibody-associated disorder; NMOSD, neuromyelitis optica spectrum disorder; OIND, other inflammatory neurological disorder. Number of patients according to the different subgroups: CNS infection (*n* = 2), CNS vasculitis (*n* = 9), MS (*n* = 58), NMOSD/MOGAD (*n* = 9), OIND (*n* = 15). ns, non-significant (*p* > 0.05). *p < 0.05. ***p < 0.001.

As shown in **Figure 3A**, elevated KFLC index strongly suggests MS diagnosis independently of the compared OIND subgroups (median of 69.1, 5.49, 1.46, and 4.05 for MS, NMOSD/MOGAD, CNS vasculitis, and OIND diagnoses, respectively; *p* < 0.001 for all comparisons). The comparison between MS and CNS infection did not seem valid, while only two patients presented with a CNS infection in our cohort. The CSF CD25 concentration (**Figure 3B**) did not seem to be effective to separate MS from NMOSD/MOGAD (median CSF CD25 of 20.5 vs. 27.6 pg/ml for MS and NMOSD/MOGAD, respectively, *p* = 0.755). Nevertheless, CSF CD25 could separate MS from CNS vasculitis (median CSF CD25 of 81.0 pg/ml, *p* < 0.001) or other types of OIND (median CSF CD25 of 51.5 pg/ml, *p* = 0.012). Finally, CSF IL-6 (**Figure 3C**) seemed to be a good biomarker to distinguish MS from NMOSD/MOGAD (median CSF IL-6 of 3.1 vs. 27.0 pg/ml for MS and NMOSD/MOGAD, respectively, *p* < 0.001) and from CNS vasculitis (median CSF IL-6 for vasculitis of 27.7 pg/ml, *p* < 0.001). However, median CSF IL-6 concentrations were not different between MS and the other OIND (*p* = 0.392).

### Cerebrospinal Fluid Kappa Free Light Chains, CD25, and IL-6 Concentrations Were Not Influenced by Age, Gender, Disease Duration, and Immune-Modifying Drug Use at Sampling

Based on the linear regression multivariate analysis model, CSF KFLC, CSF CD25, and CSF IL-6 concentrations were not influenced by age (*p* = 0.423, 0.508, and 0.891, respectively), gender (*p* = 0.840, 0.564, and 0.072, respectively), immune-modifying drug use at sampling (*p* = 0.906, 0.530, and 0.215, respectively), or disease duration (*p* = 0.0931, 0.163, and 0.126, respectively). The only factor associated with elevated CSF KFLC was MSARD diagnosis (*p* < 0.001 when compared to NIND group, and *p* = 0.001 when compared to the OIND group as reference), and the only one associated with elevated CSF CD25 and IL-6 was OIND diagnosis (*p* = 0.018 for CD25 when compared to MSARD as a reference, and *p* = 0.003 for IL-6 when compared to MSARD as reference). All data are shown in **Table 5**.

**Table 5:** Identification of clinical and demographic data influencing CSF KFLC, CSF CD25, and CSF IL-6 concentrations by linear regression multivariate analysis.

	CSF KFLC* n* = 174		CSF CD25* n* = 174		CSF IL-6* n* = 174	
	β coefficient [IQR]	*p*-Value	β coefficient [IQR]	*p*-Value	β coefficient [IQR]	*p*-Value
Age *Risk for each 1 year increase*	0.024 [−0.035; 0.083]	0.423	0.316 [−0.625; 1.260]	0.508	−0.071 [−1.100; 0.953]	0.891
Gender *Reference: women*	0.24 [−2.07; 2.54]	0.840	−7.76 [−34.23; 18.72]	0.564	40.53 [−3.61; 84.66]	0.072
Disease duration Risk for each 1 month increase	−0.04 [−0.08; 0.01]	0.093	−0.20 [−0.48; 0.08]	0.163	−0.42 [−0.97; 0.12]	0.126
Immune drug ongoing at sampling *Reference: yes*	0.11 [−1.71; 1.92]	0.906	11.52 [−24.59; 47.63]	0.530	45.32 [−26.6; 117.23]	0.215
Diagnosis *Reference: MSARD*						
NIND group	−5.53 [−7.53; −3.54]	<0.001	−7.24 [−17.04; 2.55]	0.146	−0.823 [−13.23; 11.59]	0.896
OIND group	−4.82 [−7.66; −1.98]	0.001	60.55 [10.58; 110.52]	0.018	93.56 [33.29; 153.83]	0.003

CSF, cerebrospinal fluid; KFLC, kappa free light chains; MSARD, multiple sclerosis and related disorder; NIND, non-inflammatory neurological disorder; OIND, other inflammatory neurological disorder.

## Discussion

Our study evaluates prospectively multiple CSF biomarkers in patients presenting for a diagnostic workup of brain white matter hyperintensities suggestive of MS. Our results suggest that activated B-cell biomarkers (OCBs or KFLC index/IF) may strongly recommend MSARD diagnosis regardless of the chosen control population. KFLC index has the advantage of being more sensitive than OCBs but suffered from less specificity. These results are consistent with previous retrospective (15, 16) and prospective (14, 22, 23) studies. We found that CIS patients may present with lower KFLC biomarkers values than MS and RIS patients. It may be explained that the 2017 McDonald criteria were applied for MS diagnosis. In doing so, all CIS patients presenting with radiological dissemination in space and positive OCBs were diagnosed as having MS. Therefore, in our cohort, most of the CIS patients presented with low intrathecal B-cell activity (negative OCBs).

We found that CSF CD25 and CSF IL-6 concentrations were lower in MSARD than in OIND. However, these biomarkers cannot rule in MSARD, while NIND patients also express low CSF CD25 and IL-6 concentrations. Nevertheless, high CSF CD25 and IL-6 could be helpful in rolling out MSARD diagnosis, while it would favor another MS-mimicking inflammatory CNS disease. Of note, elevated CSF CD25 presents the highest positive predictive value for OIND diagnosis, more than low KFLC index or negative OCBs. However, CSF CD25 lacks diagnostic performance in separating MSARD from NMOSD and MOGAD, whereas IL-6 seems to be an effective tool in such situations. This is why we think that CSF CD25 and CSF IL-6 should both be used in practice. Moreover, CSF KFLC, CD25, and IL6 concentrations were not influenced by age, gender, disease duration, or immune treatment used during sampling. This point is important, while diagnostic biomarkers need to be efficient at any time of the diagnostic workup.

Our results are consistent with previously published data, showing that a high KFLC index or KFLC IF is associated with MS diagnosis (14–16, 22, 24). KFLC has the advantage, compared to OCBs, in quantifying CSF B-cell activity. This is an important point to consider, while it has been shown, on pathological brain analysis, that MS patients present higher amounts of activated B cells than other inflammatory CNS disorders (25). However, many different KFLC index cutoff values were published to assess intrathecal immunoglobulin synthesis (i.e., KFLC index cutoff range from less than 3 to more than 10) (26, 27). This discrepancy could be explained by the heterogeneity of the different control populations, while many inflammatory CNS disorders may have an intrathecal B-cell activity. Therefore, as suggested by our study, cutoff values of KFLC biomarkers should be different depending on the suspected underlying MS-mimicking disorder, to avoid misdiagnosis.

Our findings agree with other retrospective studies that found an increased concentration of CSF IL-6 in NMOSD (17, 28, 29) and MOGAD (17, 30) compared to MS. Added to our results, these findings suggest that CSF IL-6 measurement may impact early diagnosis, while cytokine measurement is easy and fast to perform as compared to aquaporin-4 or MOG antibody, and may guide early therapeutic action, in suspected NMOSD/MOGAD patients. Moreover, tocilizumab, an IL-6 receptor blockade therapy, has shown promising efficacy in NMOSD (31–33) and has been reported to be effective in relapsing steroid-dependent MOGAD (31, 34), reinforcing the impact of the IL-6 pathway in these diseases. In contrast, CSF CD25 could not separate MSARD from NMOSD and MOGAD, as it has already been suggested in two previously published retrospective studies (17, 35). However, because of its high positive predictive value for OIND diagnosis, elevated CSF CD25 should be used as a non-specific MSARD red flag. Even if CSF CD25 is not associated with a specific disease in our heterogeneous cohort, it could be an exciting tool in neurosarcoidosis (18), bacterial meningitis (18), or CNS lymphoma (18, 35, 36).

**Figure 4:**
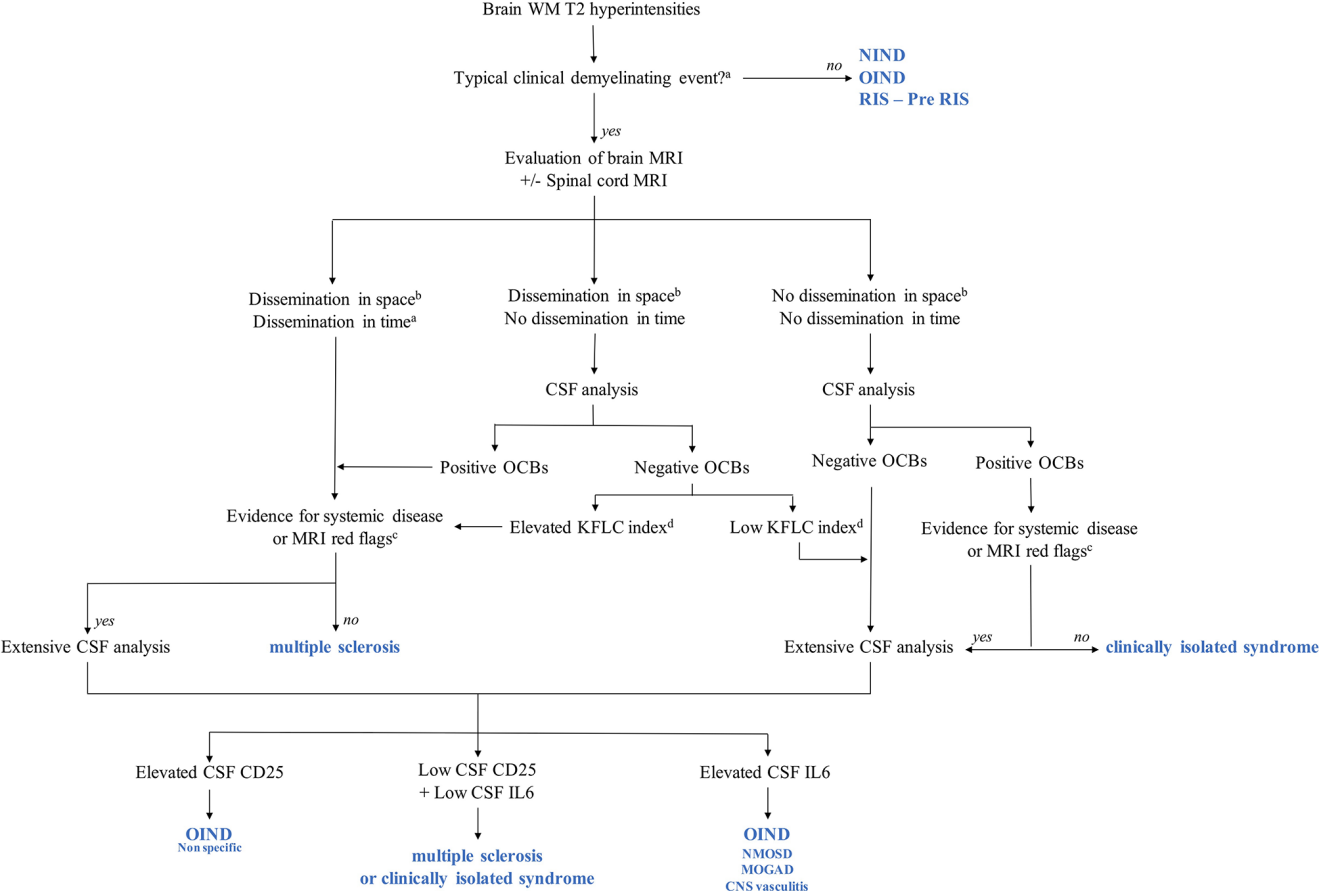
Multiple sclerosis (MS) diagnostic algorithms including KFLC index, CSF CD25, and CSF IL-6. MOGAD, myelin oligodendrocyte glycoprotein antibody-associated disease; NIND, non-inflammatory neurological disorder; NMOSD, neuromyelitis optica spectrum disorder; OIND, other inflammatory neurological disorder; Pre-RIS, radiologically isolated syndrome with one or two specific dissemination in space criteria; RIS, radiologically isolated syndrome; WM: white matter. ^a^According to reference (1). ^b^According to reference (37). ^c^According to reference (38). ^d^In our study, the KFLC index cutoff was 13.1. This cutoff is specific to our cohort and should not be used in daily practice, while each MS tertiary center should determine its threshold values. KFLC, kappa free light chains; CSF, cerebrospinal fluid.

Nonetheless, in clinical practice, a spinal tap is not performed in all suspected MS patients, while MS diagnostic criteria are based on the presence of a typical demyelinating event and MRI presentation (1). However, CSF analysis is often performed, while identifying OCBs is a key point to ensure MS diagnosis and avoid misdiagnosis. Moreover, our results show that extensive CSF analysis could help etiological diagnosis in many complicated cases. Importantly, none of the NIND patients in our cohort experienced a typical clinical demyelinating event, reinforcing the importance of clinical presentation in fulfilling MS criteria and identifying red flags for MS diagnosis. According to these findings and the current recommendations for MS diagnosis (1, 37, 38), we provide an MS practical diagnostic algorithm for patients presenting with brain white matter hyperintensities suggestive of MS (**Figure 4**).

Our study suffers from several limits. First, being a monocentric study, our results need to be confirmed by others, even if these results are consistent with multiple retrospective data. Second, our cohort’s small size and heterogeneity, particularly in the OIND group, do not permit us to conclude on the effectiveness of these biomarkers for the different MS-mimicking diseases. However, it allows figuring out which biomarker may help in rolling in or rolling out MSARD. Third, it would have been interesting to measure serum IL-1β, CD25, IL-6, and IL-10 to calculate cytokine indexes, but our routine diagnostic workup of white matter hyperintensities does not include these analyses. Nevertheless, this study is pragmatic, evaluating these biomarkers prospectively in daily practice for the diagnostic workup of suspected MS. We think that these results will increase the etiological diagnostic accuracy of such patients.

## Conclusion

In patients presenting for a diagnostic workup of MRI white matter hyperintensities, elevated CSF activated B-cell biomarkers such as KFLC index or KFLC IF strongly suggest MSARD. In contrast, elevated CSF IL-6 and CD25 suggest another inflammatory-mimicking disease. These findings need to be confirmed in other prospective cohort studies within larger samples.

## Data Availability Statement

The raw data supporting the conclusions of this article will be made available by the authors, without undue reservation.

## Ethics Statement

Ethical review and approval were not required for the study on human participants in accordance with the local legislation and institutional requirements. The patients/participants provided their written informed consent to participate in this study.

## Author Contributions

ML and CL-F designed the study, collected the data, performed the data analysis, and drafted the manuscript. CL, LM, MC, and SB helped in designing the study, data collection, preparation of the manuscript, and review for intellectual content. VB and BS-P performed the biomarker analysis and helped in reviewing the manuscript for intellectual content. All authors contributed to the article and approved the submitted version.

## Conflict of Interest

The authors declare that the research was conducted in the absence of any commercial or financial relationships that could be construed as a potential conflict of interest.

## Publisher’s Note

All claims expressed in this article are solely those of the authors and do not necessarily represent those of their affiliated organizations, or those of the publisher, the editors and the reviewers. Any product that may be evaluated in this article, or claim that may be made by its manufacturer, is not guaranteed or endorsed by the publisher.

## Supplementary Material

Supplementary Figure 1 Flow chart. CNS, central nervous system; MS, multiple sclerosis; MSARD, multiple sclerosis and related disorder; OIND, other inflammatory neurological disorder; WMH, white matter hyperintensities.

Supplementary Figure 2 Distribution of KFLC biomarkers into RIS, CIS, and MS subgroups. CIS, clinically isolated syndrome (*n*=22); MS, multiple sclerosis (*n*=58); RIS, radiologically isolated syndrome (*n*=8); ns, non significant ***p < 0.001.

Supplementary Figure 3 Distribution of CSF CD25, IL6, and IL10 into RIS, CIS, and MS subgroups. CIS, clinically isolated syndrome (*n*=22); MS, multiple sclerosis (*n*=58); RIS, radiologically isolated syndrome (*n*=8). ns, non significant *p < 0.05.

Supplementary Figure 4 ROC curves of KFLC index, KFLC IF, CSF CD25, IL6, and IL10.[(**A**) ROC curve that separate MSARD from NIND (*n*=141)]. Panel **(B)** ROC curve that separate MSARD from OIND (*n*=123).
