# Supplementary figures and images for: Kappa Free Light Chains, Soluble Interleukin-2 Receptor, and Interleukin-6 Help Explore Patients Presenting With Brain White Matter Hyperintensities
Source: Front Immunol. 2022 Mar 25;13:864133. doi: 10.3389/fimmu.2022.864133 (PMC8990749; doi:10.3389/fimmu.2022.864133)

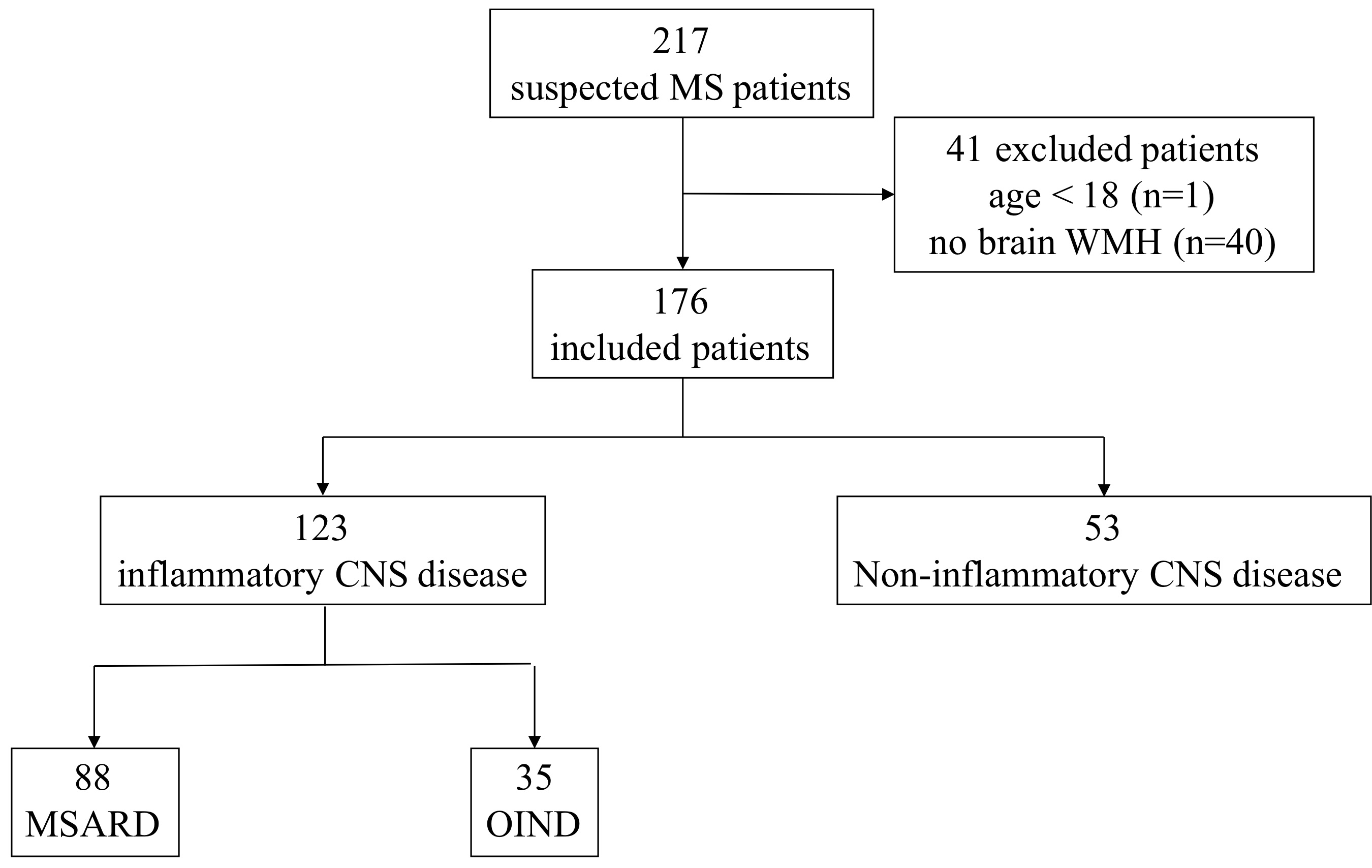

Supplement: Supplementary Figure 1 — Flow chart. CNS, central nervous system; MS, multiple sclerosis; MSARD, multiple sclerosis and related disorder; OIND, other inflammatory neurological disorder; WMH, white matter hyperintensities. [file Image_1.jpeg]

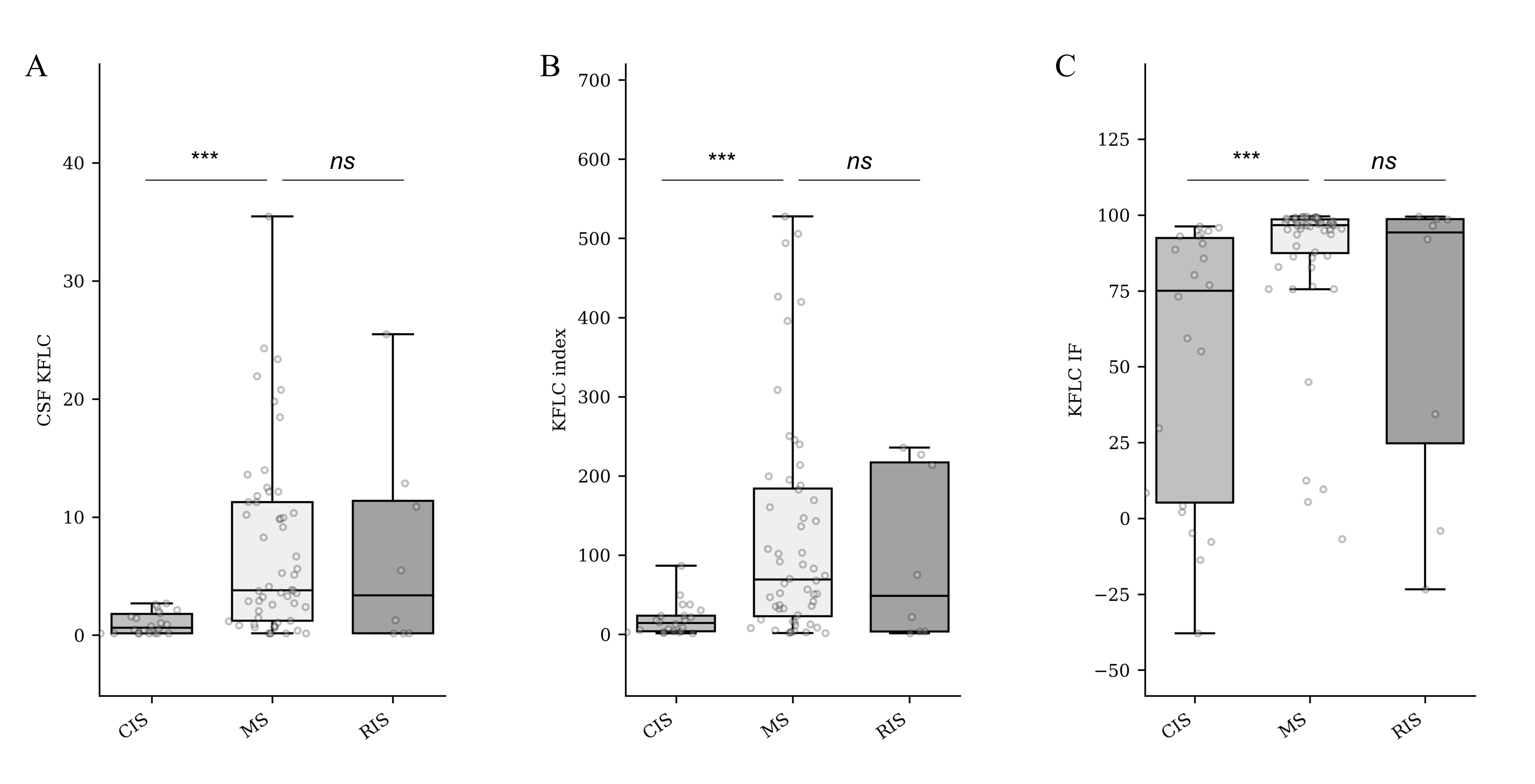

Supplement: Supplementary Figure 2 — Distribution of KFLC biomarkers into RIS, CIS, and MS subgroups. CIS, clinically isolated syndrome (n=22); MS, multiple sclerosis (n=58); RIS, radiologically isolated syndrome (n=8); ns, non significant ***p < 0.001. [file Image_2.jpeg]

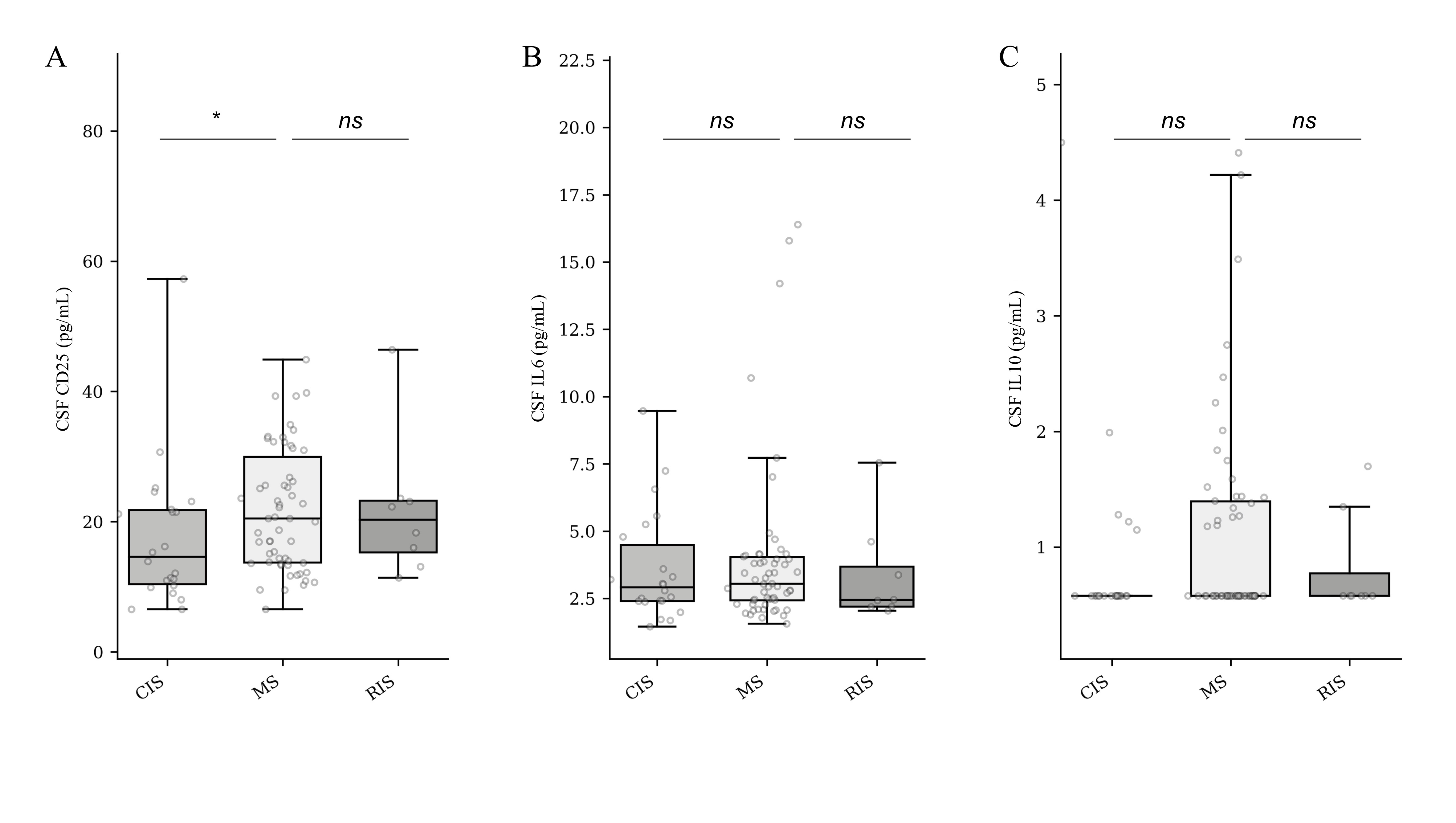

Supplement: Supplementary Figure 3 — Distribution of CSF CD25, IL6, and IL10 into RIS, CIS, and MS subgroups. CIS, clinically isolated syndrome (n=22); MS, multiple sclerosis (n=58); RIS, radiologically isolated syndrome (n=8). ns, non significant *p < 0.05. [file Image_3.jpeg]

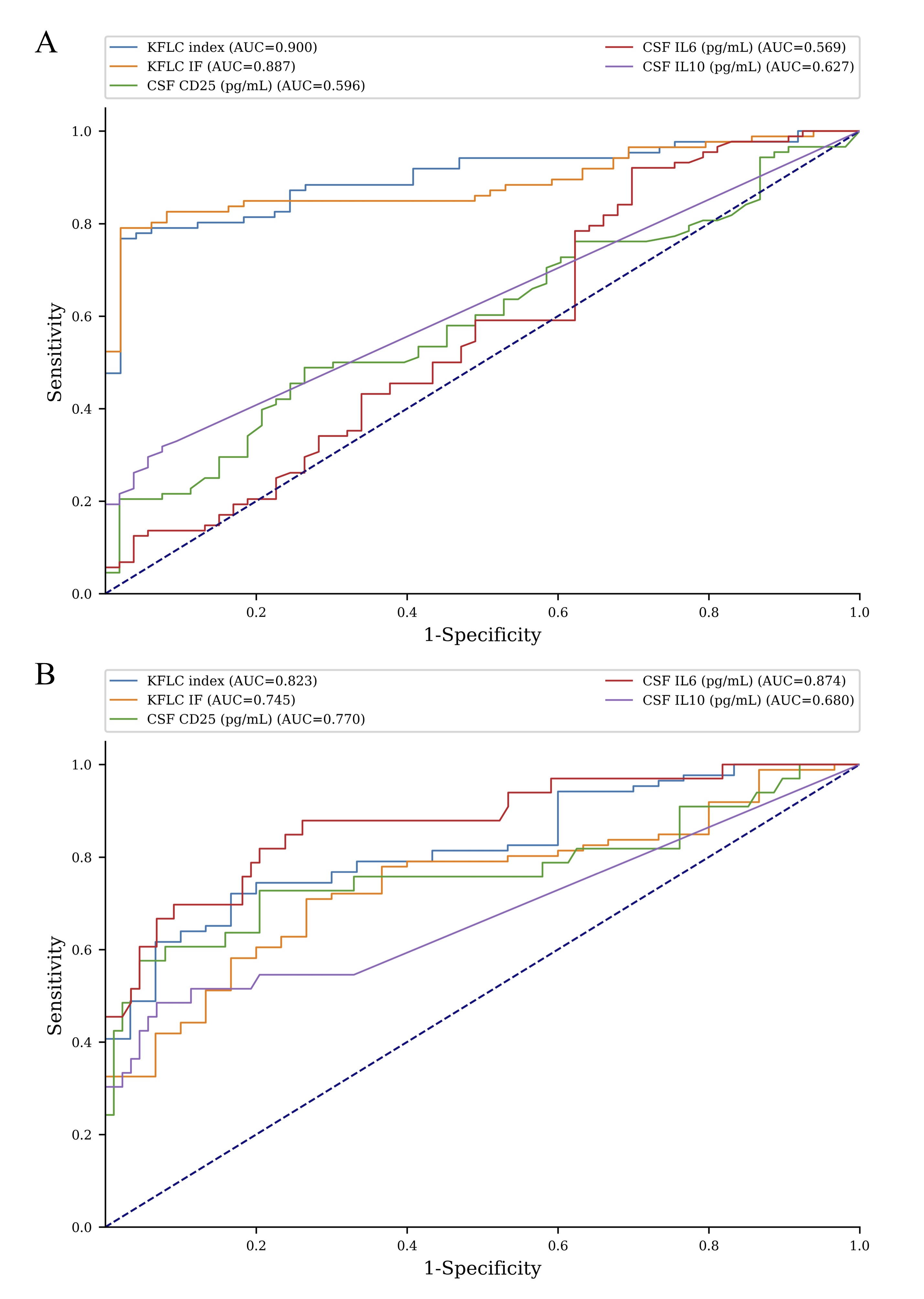

Supplement: Supplementary Figure 4 — ROC curves of KFLC index, KFLC IF, CSF CD25, IL6, and IL10.[(A) ROC curve that separate MSARD from NIND (n=141)]. Panel (B) ROC curve that separate MSARD from OIND (n=123). [file Image_4.jpeg]
